# Subjective Well-Being of Professional Females: A Case Study of Dalian High-Tech Industrial Zone

**DOI:** 10.3389/fpsyg.2022.904298

**Published:** 2022-07-05

**Authors:** Yuqing Zhang, Ya Gao, Chengcheng Zhan, Tianbao Liu, Xueming Li

**Affiliations:** ^1^School of Geography, Liaoning Normal University, Dalian, China; ^2^Dalian No. 8 Senior High School, Dalian, China

**Keywords:** professional females, intra-household life, extra-household life, life satisfaction, emotional cognition

## Abstract

The education level and social participation of contemporary Chinese women have reached their historical peak; work is fast becoming the dominant theme of their lives. However, influenced by traditional attitudes, women are still expected to undertake the main family care tasks, thus, facing dual constraints of family and work, which seriously affect their life happiness. Based on the theory of subjective well-being and feminist geography, this study used the questionnaire survey and in-depth interview results of professional females in Dalian High-tech Industrial Zone as basic data to explore the life satisfaction and emotional cognition in intra- and extra-household life of professional females (Professional females: In this study, they are the women who have received formal education and currently have full-time and steady job (including regular employees in the national systems and those who have signed labor contracts with labor units).). The following results were obtained: (1) Most professional females reported higher life satisfaction in intra- rather than extra-household life, and it varied with individual attributes, reflecting the internal differences among them. (2) The positive emotions of professional females came from the company of family and friends in intra-household life, and satisfaction with the working environment and treatment in extra-household life. (3) The negative emotions came from the pressure of “marriage,” “birth,” and other traditional concepts in intra-household life. In extra-household life, it came from the health problems caused by working stress, interpersonal problems and gender inequality in the workplace, and the anxiety of age and future career development. Therefore, this study committed to revealing the living status and subjective feelings of contemporary professional females in China, hoping to improve women’s life quality and enhance their life happiness from a theoretical and realistic perspective.

## Introduction

With China’s sustained economic development, its residents are paying more attention to the improvement in their quality of life and the pursuit of happiness (Davey and Rato, 2012; Easterlin et al., 2012). Subjective well-being (SWB) has thus become a comprehensive indicator of social development (Cummins, 2018; Wang et al., 2020; Pleeging et al., 2021). It is an individual’s overall perception of their quality of life (Veenhoven, 2004; Pleeging et al., 2021), including lifew satisfaction and emotional cognition (Appleton and Song, 2008; Wang and Wang, 2015, 2016), where the former reflects the individual’s overall assessment and cognition of life stability, and the latter expresses the short-term emotional experience, including active and negative feelings (Diener et al., 1999; Dos Santos et al., 2019). SWB has a long history in the fields of psychology, economics, and sociology, which have expanded research on the subject matter mainly from the perspectives of economic benefits and psychological implications. Meanwhile, geography focuses on discovering the relationship between happiness and space, including the spatial differences of SWB, the perception of happiness caused by differences in climate and natural geographical environment, the influence of urban spatial layout and built environment factors on SWB, and the like (Wang and Wang, 2015; Dang et al., 2018).

At present, many countries are discussing SWB based on their own national conditions and social environment. With the concept’s popularization, the subject matter and themes of its research have become more abundant, specific, and in-depth. Western studies mostly focus on the measurement, evaluation, and influencing factors of SWB (Ballas, 2013). Early studies believed that SWB stems from internal states such as fixed thinking and personality rather than external interference. However, later studies began investigating the impact of regional environmental and natural factors such as environmental pollution and biodiversity on SWB (Mavoa et al., 2019). With the continuous deepening of research, researchers gradually realized that individual SWB is affected by a combination of many factors, such as age, gender, health status, and other personal or family factors (Steptoe et al., 2015; Lim, 2020), and thus emphasized the connection between people and society (Chung and Hahn, 2020; Hsieh and Li, 2021). Consequently, the impact of social support, social cohesion, social capital, sense of belonging, gender equality, and political participation on SWB has gradually become the focus of current research (Atkinson et al., 2020; Salahodjaev et al., 2020).

In the 1980s, the study of SWB in China gradually started based on the introduction of relevant foreign research results, gradually combining China’s actual conditions and the influence of respective academic backgrounds, such as the residents’ income, environmental quality, social security, and traffic convenience, on individual SWB (Guodong et al., 2005; Yunxiao et al., 2014; Jiangjie and Bindong, 2016), to construct a measurement system for the residents’ SWB. With the recent improvement in residents’ living standards, the measurement of individual SWB has become an important inspiration for the formation of “happy cities” and “smart cities” (Wang and Wang, 2015). Therefore, in addition to discussing the impact of social background on individual SWB, scholars have also been particularly interested in the impact of urban and community formation on SWB in the context of rapid urbanization (Liu et al., 2019; Zhou et al., 2019), the urban–rural differences in SWB (Dang et al., 2018), the role of participation in leisure and tourism activities in improving SWB (Wu et al., 2019), and the impact of life pressure on individual well-being (Zhang and Zhang, 2016).

Integrating relevant domestic and foreign research reveals the key research objects of SWB to be special groups such as floating populations (Liu et al., 2017, 2018), children (Tomyn et al., 2017; Cho, 2018), and older people (Zhou et al., 2019; Appau et al., 2020). However, happiness researchers have largely ignored the other half of the human race: females. Women’s emotional experiences are more sensitive and they have clear gender advantages in emotional feedback and cognitive judgment. With the continuous improvement of Chinese women’s social status and education level, professional females, as one of the main forces in China’s economic structure, also play major roles in family life. Being the primary handler of household chores (Jiaming et al., 2017; Chen, 2020; Yuchen, 2021), they face both domestic and working constraints (Lee, 2002; Yuqing and Chengcheng, 2019; Yuqing et al., 2021), their feelings of happiness are more diversified, and can comprehensively reflect the reality of contemporary Chinese women’s life; however, the SWB of professional females is rarely mentioned. As anthropology, sociology, politics, history, and other disciplines consider feminism an important research paradigm, trying to view and reflect the social status of contemporary women jointly established by the state, market, and society under specific space-time coordinates (Henriques and Garcia, 2022), feminist geography relies on its unique comprehensive characteristics and powerful spatial analysis ability to describe and probe the female activities and characteristics in various fields, so as to study the interaction between women and space and to explore the role and mechanism of women in the shaping of urban space and place. It is, therefore, very suitable for lending theoretical support to female SWB research. However, in the current system of research, few studies have linked feminist geography with women’s SWB from the perspective of the interaction between intra- and extra-household life. Previous research on residents’ SWB has usually focused on the impact of a single activity or evaluation mechanism, ignoring the individual emotional duality of positive emotions and negative attitudes and lacking a combination of the two for a more comprehensive evaluation. Moreover, the SWB of residents is rarely analyzed from the perspective of rational thinking, and it is even more difficult to combine quantitative research with qualitative research. Therefore, this study conducted field research on professional females in Dalian High-tech Industrial Zone, used questionnaire survey and in-depth interview data, combined quantitative research with qualitative research, and applied feminist geography and resident happiness theories to analyze the SWB of professional females in contemporary China. We attempt to enrich the SWB research of female residents from the perspective of feminist geography, comprehensively demonstrating the happiness acquisition of professional females under the dual constraints of life intra and extra home. In doing so, the study aims to address the following research questions: (1) What factors affect professional females’ life satisfaction and emotional cognition under different life conditions intra- and extra home? (2) What is the ideal life for professional females? and (3) What do they expect in intra- and extra-household life? Most importantly, we try to apply feminist geography theory to the practice of women’s daily life study, shedding light on contemporary Chinese women’s studies and even gender studies.

## Theoretical and Realistic Basis

### Subjective Well-Being Theory Construction of Professional Females

Based on the theory of feminist geography and residents’ SWB and considering the particularities of professional females, this study constructed a model of their life satisfaction and emotional cognition. The ontological problem faced by feminist geography is the gender oriented social spatial structure (Massey, 1994; Sun et al., 2019). It is not only manifested in the physiological gender differences, but also in the cultural and psychological gender differences through employment, health, education, interpersonal relations, assets, and other fields (Li et al., 2020).

This study classifies the evaluation index of professional females’ life satisfaction into five aspects: health, living environment, leisure activity, career, and salary satisfaction. (1) Health satisfaction refers to women’s health status as the basic standard for measuring the level of social progress and their development. Feminist geography believes that women’s health is not only affected by internal factors, but also by public facilities (Huang and Gu, 2003; Zheng and Zhen, 2010), commuting time (Roberts et al., 2011), leisure and entertainment time (He et al., 2019), travel mode (Dyck, 2006), objective environment (Zhang L. et al., 2019), family (Broom, 2014), and other factors. (2) Regarding living environment satisfaction, the development of human living space is accompanied by the transformation of women’s behavior and role status. Traditional gender roles require women to focus on family, which leads to their being more obedient and attentive to the needs of the husband and the family when it comes to choosing residence, employment, and even commuting (Zhou, 2014). However, for Chinese families with a high proportion of dual employees, women wield greater control in deciding where to live (Zhou, 2014; Ta and Liu, 2017). Family responsibilities such as housework, eldercare, and childcare also make women pay more attention to the infrastructure layout of the living environment (Chen, 2005), and women’s power within the family is higher than that of men (Feng et al., 2015; Zhang and Zhang, 2016). Therefore, residence satisfaction can characterize the daily life rights of Chinese women. (3) Regarding leisure activity satisfaction, the positive effect of leisure on SWB and its moderating effect on mental status have been confirmed and affirmed by scholars (Heintzman and Mannell, 2003; Baumeister et al., 2013; Wang and Sun, 2019; Zhang Y. et al., 2019). It is also relevant to discuss women’s leisure from the perspective of geography. Professional females take care of their families amid daily tense working environments, and their satisfaction with leisure activities can reflect relief from their daily stress. (4) Regarding career satisfaction, women’s employment and other related issues have always been the traditional field of feminist geography research. In addition to personal factors, external factors such as human and employment environment (Sun et al., 2018; Fu et al., 2020), urban transportation (Liu and Noback, 2011; Chung and Hahn, 2020), and family care (Noback et al., 2013; Bičáková, 2016) also have an important impact on women’s labor participation. Therefore, professional females’ satisfaction with their career can reflect their evaluation of the overall employment environment. (5) Salary satisfaction in the context of social development reveals that the external inequality experienced by women is largely reflected in the gender difference in salary (Boyer and England, 2008; Yao et al., 2017; Semenza et al., 2021), even though the labor participation rate of Chinese women is higher than the world average, the gender gap in pay still exists. Therefore, salary satisfaction can be used to measure gender equality at the workplace.

In terms of emotional cognition, feminist geography has since the beginning of the 20th century shifted from the perspective of geographic and spatial aspect to the cognition of female subjective emotions (Kwan, 2007; Otrel-Cass, 2016), in that it is no longer completely attached to the fixed concept of “place” (Sun et al., 2019). Emotions include not only the emotional connection of human beings with others in social life (Bondi, 2005), but also the changes in mental state (positive and negative), keeping in mind the cultural and demographic differences in emotional experience (Diener et al., 2003). For professional females in China, the indicators that reflect their emotional cognition level are mainly reflected in their career, family, daily life, and psychological activities. This study explores the emotional cognition of professional females from the two aspects of positive and negative emotions. Positive emotion is the main source of pleasure, including good health, emotional comfort, participation in leisure activities, career development, and salary boost. (1) Good health: many feminist researchers associate women’s health with social development (Moen et al., 1992; Zhang L. et al., 2019). Women’s evaluation of their own health status is also an important criterion that reflects their physical and psychological condition and quality of daily life. (2) Emotional comfort: women’s cognition of interpersonal relationships and family connections in life can express their perception of happiness (Sharp, 2009), with emotional exchanges with others also having an invisible comforting effect on them. (3) Participation in leisure activities: as mentioned above, professional females’ participation in leisure activities can reduce the pressure of work and life and enhance their sense of happiness (Xu and Chai, 2011; Roeters et al., 2016). (4) Career development: gender inequality in career development has been confirmed by multiple practical and theoretical studies (Lin and Junfeng, 2003; Guangqiang, 2014; Aslan Akman, 2021), therefore, promotion of professional females can, to a large extent, represent the gender development of the industry, which may affect their emotions. (5) Salary boost: as mentioned above, salary satisfaction is an important indicator of professional females’ SWB, and an increase in income will improve their positive emotions. In the contemporary fast-moving urban life, the sources of residents’ negative emotions vary. The evaluation and outlet analysis of residents’ negative emotions are also important aspects of happiness research. According to the foregoing theoretical basis of professional females’ life satisfaction and positive emotions, a reverse analysis of the root causes of their negative emotions can be divided into: personal, work, family, and financial pressures.

**FIGURE 1:**
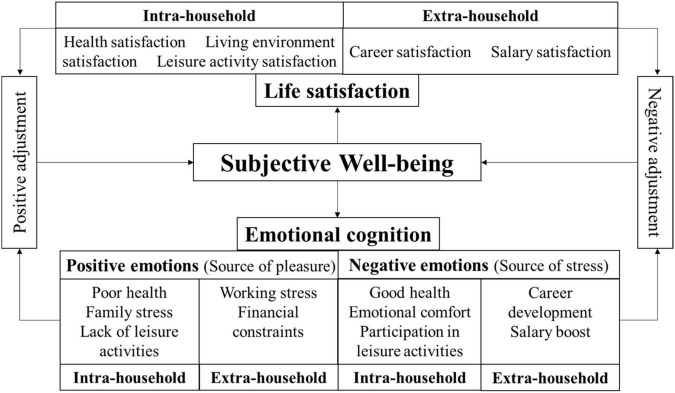
The model construction of SWB.

Based on the abovementioned theoretical analysis, a model was constructed for the professional females’ SWB (Figure 1).

### The Living Actuality of Contemporary Chinese Professional Females

#### The Intra-household Power Relations

Feminist geography, time geography, and other theories explain “home” as important “pockets of local order” (Lenntorp, 2004) for residents’ daily activities. There is a significant gender difference in activity participation within home, with many scholars suggesting that women are subject to more fixed constraints than men (Chen et al., 2019). Consequently, there are gender differences in behavior pattern, activity participation, time use, and other time-space behavior patterns. In family life, especially in married nuclear families with children, men tend to spend more time at work, and women are more involved in housework and childcare (Kwan, 2000; Ettema and van der Lippe, 2009). Most of the shopping activities in family are also undertaken by women. The family model, “men primarily as breadwinners and women as homemakers” (Yoon, 2010) is considered as the “natural order” (Yuqing et al., 2021), exacerbating gender inequalities in daily life and increasing the time poverty that women face. Scholars have also proposed and verified the hypothesis of “Household responsibility” (McLafferty and Preston, 1991; Johnston-Anumonwo, 1992), that women in employment are expected to have more family and childcare responsibilities than men, putting them under greater time constraints and causing them to choose occupations with shorter commutes. This, however, leads to poor career stability and split work time for women, forcing them into part-time jobs or even completely withdrawing from the labor market (Yuqing et al., 2021). In addition, men in the family have more power over the choice of the location of the residence and housing types (Bergan et al., 2021). Many studies have also found that a large part of women’s perceived happiness comes from pleasant interactions with family members and leisure time enjoyed away from home and work (Brethel-Haurwitz and Marsh, 2014; Kekäläinen et al., 2020).

In contrast to western countries, family relationships in China and some parts of the East Asian Chinese world emphasize respect for elders and observance of filial piety (Qian and Wuqing, 2012; Ying et al., 2013). Chinese women’s rights in the family are changing constantly. In feudal times, women were required to abide by the “three subordinate” rules from birth: “be obedient to the father before marriage, be obedient to the husband after getting married, and be obedient to the son after the husband’s death” (Xiaojiang, 2018), indicating that a woman’s whole life is subordinate to a man, at different times in life. At that time, women did not have their own dominant status and could not make a living independently in society, not to mention happiness, quality of life, and other self-life value measurement significance. This historical period was very long, almost running through the whole history of ancient Chinese civilization, and engendered a strict gender system (Xiaobao, 2019), including spawning many vices against women. However, with the liberation of new China, and under the agitation of the global feminist trend of thought, the quality of life and education of Chinese women have improved significantly, enabling them to reinvent themselves. Nowadays, Chinese women are more independent, intelligent, and confident, exerting their strength and charm in different fields of social life together with the men; gender equality has become one of China’s most important state policies. However, many new problems have emerged in modern Chinese family life. As a researcher at the Chinese Academy of Social Sciences once put it bluntly: no matter how talented or accomplished a woman is, if she is unwilling or unable to be a tender lover, a caring wife, a loving mother, her beauty to me is greatly diminished. Referring to the marriage of female PhD students in China, a member of the Chinese People’s Political Consultative Conference infamously remarked: if a girl has not “sold” herself for more than twenty years, from a romantic point of view, pursuing her PhD degree will not increment her value, instead, it will devalue her. A famous geographer in China also once said: only when women learn geography well, can the whole country learn geography well, because the education of children in the family is the responsibility of women. Such views are inevitably attacked in Chinese society today, however, in daily life, phrases such as “graceful in the drawing room and skillful in the kitchen” are still commonly used to praise women who have achieved a perfect balance in their life at home and outside. That is, even though the status of Chinese women has risen to the highest level in history, their societal responsibilities and obligations have not decreased, hence, they face the double constraints of work and family (Chen et al., 2019; Yanxi et al., 2021). Therefore, many scholars believe that in the context of the continuation of the traditional concept that “man goes out to work while woman looks after the house,” and the large-scale employment of Chinese women, the division of roles within the family is not equal, but more traditional and even exhibits a strengthening trend (Chunhua et al., 2011). For example, in intra-household life, married women with children are under pressure to raise children, they may have higher depressive symptoms when under parenting stress, leading to lower marital satisfaction (Dong et al., 2022). Although the happiness of women in Chinese family life is now generally higher than that of men, from the perspective of longitudinal time, the fast pace of life, and the increase of family responsibility burden, the happiness of women tends to decline (Roeters et al., 2016; Dang et al., 2018).

#### The Extra-Household Power Relations

Under the trend of postmodernism, in the research system of the field of humanities, research based on gender perspective is gaining more and more attention, to strike back the rationalization and regularization of the construction of male-dominated power space under the orientation of essentialism and universalism that ignores and devalues femininity (Massey, 1994). In addition to the natural nature of individual identity, gender should be viewed as a social relationship. The difference between men and women is social because in social life, gender is a field of social inequality, where the struggle for social change and justice and against the privileged takes place (Ortner, 1972; Tivers, 1978). As McDowell points out, the ontological problem faced by feminist geography is the problem of gendered social space structure (McDowell, 1993). This gendered social space structure is a hierarchical binary social space structure, which is male-dominated (Massey, 1994). This leads to a series of gendered dualistic representations in geographical space: male represents public space, external space, work space, production space, power space, independent space, and so on, while female represents private space, inner space, reproduction space, leisure space, entertainment space, consumption space, dependence space, weak power space, and so on. Among these spaces, masculine spaces are productive and dominant, while feminine spaces are reproductive and dependent (Daphna-Tekoah et al., 2021). This state is obviously unbalanced and not conducive to social progress.

The most important field for extra-household activities is the work field, and as more and more women are involved in all forms and domains of workplace, women’s commuting characteristics and their use of transportation are key research areas. Studies have found that long commutes have a greater negative impact on women’s SWB. In the relatively broad public space, except the workplace, studies have found that women are actively or passively excluded from the public space (Chen et al., 2021; Tamboukou, 2021). Chinese women also encounter various explicit and implicit problems in contemporary urban production activities and daily life. From the old patriarchal power, husband’s power, and other familism to women’s moral constraints, the strategies of gender discrimination in the context of “World Factory” in the early stage of reform and opening up of China (Chen and Chen, 2021; Yuchen, 2021), and the masculinity of various public service facilities and public spaces under the male-dominated thinking in urban planning (Zhou, 2014), all are causing women in cities to encounter inequality one way or another. For women at work, although the female labor force participation rate in China is much higher than the world average at over 62% (The World Bank., 2020), implicit discrimination in the workplace is gradually manifested; gender discrimination in recruitment, promotion, salary, and other aspects still exists. Thereby, the pressure on women at work is much higher than that on other groups, which seriously affects their daily life experience (Lin et al., 2020). Although these practical problems need to be revised and improved from a theoretical research and public policy-making perspective, most of the above discussion comes from the observation, theoretical description, and analysis of women’s behavior state. With the continued improvement of Chinese women’s education level and social status, their personal awareness, competence, and self-empowerment also continue to grow, causing large number of professional females with advanced education, senior positions, and high salaries to emerge. They choose late marriage and late childbirth, even preferring to stay unmarried and childless, so they have fewer family constraints (Yuqing et al., 2021). Single women in this state exhibit temporal and spatial behavior patterns very similar to single men, their lives are much richer, and they report higher life satisfaction. Thus, the sources of SWB of women in different life conditions, different family living environments, and different roles are more diverse, which leads to more diversified subjective perceptions. Therefore, the first-hand investigation of professional women from micro and realistic perspectives can more truly obtain their actual living conditions and understand their subjective feelings.

## Empirical Investigation

### Study Area

**FIGURE 2:**
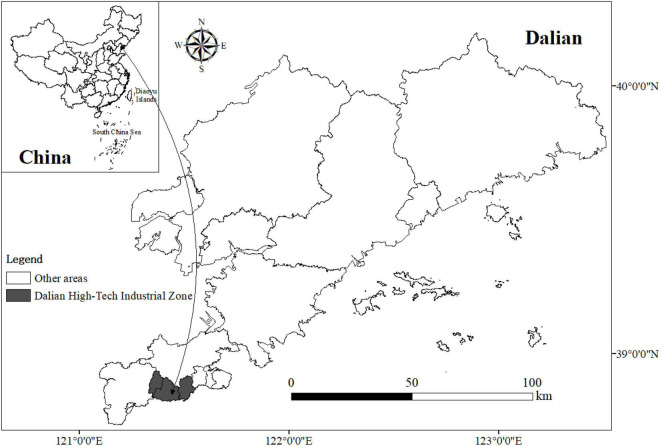
Study area.

The study area of the research is Dalian High-tech Industrial Zone in Liaoning Province (Figure 2). It’s located in the southwest of Dalian, covering an area of 153 km^2^ with a coastline of 41.6 kilometers and jurisdiction over three streets: Lingshui, Longwangtang, and Qixianling. As of January 2022, there are about 306,800 permanent residents and an employment rate of over 70% (Dalian High-tech Industrial Zone, 2022). Dalian High-tech Industrial Zone is the first batch of national high-tech industrial zones and national digital service export bases established in March 1991. It is an important residential area and employment core area in Dalian, thus, for research on professional females, it possesses high typicality and representativeness.

### Methodology and Data Collection

This study used questionnaire survey and in-depth interview data and combined quantitative research with qualitative research, from the aspects of theoretical model and subjective feelings to analyze the SWB of professional females of contemporary China.

The questionnaire data were obtained from a two-week electronic questionnaire survey conducted by the research team in April 2020. Three hundred and fourteen professional females were selected from Dalian High-tech Industrial Zone by random sampling, 291 questionnaires were returned, resulting in a response rate of 92.68%. To ensure the reliability and validity of the questionnaire results, 266 valid questionnaires were finally selected after careful verification, re-examination, and elimination of unqualified questionnaires. Finally, 91.41% of the responses were considered valid for the study.

The questionnaire collected the following information about professional females: personal and family information, spatial behavior characteristics, and time utilization. Likert scale was used to measure the five indicators of life satisfaction. Respondents were asked to choose from five options ranging from “very dissatisfied” to “very satisfied” based on their actual feelings, with the score assigned according to the number and proportion of the questionnaire results, scoring from low to high (1–5 points). The affective cognition of professional women was investigated by the ranking method. Five sources each of positive and negative emotions were listed and respondents were asked to rate the various sources separately, with the scores summarized and ranked.

The descriptive analysis results of the basic information of the respondents were collated (Table 1). Most of the surveyed professional females were aged between 25 and 44 years (83.84% of the total). Most of them had a bachelor’s degree (78.95%) and Dalian census registration (69.17%), unmarried women were as numerous as married women (49.25% vs. 50.75%), most married women had children (70.37%), and most professional females’ monthly income ranged between 2001 and 10,000 RMB (87.97%). The descriptive results of professional females’ SWB are shown in the last three columns of the table. Their life satisfaction scores ranged from 3.16 (Foreign Enterprise female employees) to 3.97 (monthly income > 10,000 yuan), and the main source of positive and negative emotion was salary boost and poor health respectively.

**TABLE 1:** Comprehensive evaluation of life satisfaction of professional females.

Category	Attribute	Variable	Number	Life satisfaction score	Primary source of emotional cognition	
					Positive emotions	Negative emotions
Personal attributes	Age	19∼24	31 (11.65%)	3.49	Salary Boost	Financial Constraints
		25∼34	156 (58.65%)	3.36	Salary Boost	Lack of Leisure Activities
		35∼44	67 (25.19%)	3.39	Good Health	Family Stress
		45∼54	5 (1.88%)	3.72	Salary Boost	Family Stress
		Over 55	7 (2.63%)	3.83	Good Health	Poor Health
	Education degree	High school and below	17 (6.39%)	3.64	Salary Boost	Financial Constraints
		Diploma or undergraduate	210 (78.95%)	3.38	Salary Boost	Working Stress
		Masters’ degree or above	39 (14.66%)	3.43	Career Development	Working Stress
The intra- household attributes	The household registration	Local census register	184 (69.17%)	3.40	Salary Boost	Poor Health
		Non-local census register	82 (30.83%)	3.41	Salary Boost	Financial Constraints
	House property rights	Own house	170 (63.91%)	3.46	Salary Boost	Poor Health
		Rental housing	96 (30.09%)	3.26	Salary Boost	Financial Constraints
	Vehicle ownership	None	106 (39.85%)	3.35	Salary Boost	Poor Health
		1	138 (51.88%)	3.41	Salary Boost	Poor Health
		≥ 2	22 (8.27%)	3.64	Salary Boost	Poor Health
	Family organization	Single	131 (49.25%)	3.40	Salary Boost	Financial Constraints
		Married without children	40 (15.04%)	3.45	Good Health	Working Stress
		Married with children	95 (35.71%)	3.38	Salary Boost	Poor Health
The extra-household attributes	Career type	Civil Servants	12 (4.51%)	3.36	Salary Boost	Working Stress
		PI Staff Members	20 (7.52%)	3.47	Salary Boost	Lack of Leisure Activities
		SoE employee	21 (7.89%)	3.72	Salary Boost	Working Stress
		FE employee	75 (28.20%)	3.35	Salary Boost	Working Stress
		PE employee	138 (51.88%)	2.99	Salary Boost	Working Stress
	Income	≤2,000 RMB	6 (2.26%)	3.26	Salary Boost	Financial Constraints
		2,001∼5,000 RMB	113 (42.48%)	3.17	Salary Boost	Poor Health
		5,001∼10,000 RMB	121 (45.49%)	3.51	Career Development	Poor Health
		>10,000 RMB	26 (9.77%)	3.97	Emotional Comfort	Lack of Leisure Activities
	Working hours	<5 h	63 (23.68%)	3.42	Salary Boost	Financial Constraints
		5–8 h	92 (34.59%)	3.38	Salary Boost	Financial Constraints
		>8 h	111 (41.73%)	3.16	Salary Boost	Working Stress
	Working-Living distance	<1 km	26 (9.77%)	3.55	Salary Boost	Working Stress
		1–5 km	106 (39.85%)	3.48	Salary Boost	Poor Health
		5–10 km	59 (22.18%)	3.31	Salary Boost	Poor Health
		>10 km	29 (28.20%)	3.31	Salary Boost	Poor Health
	Commuting hours	<15 min	46 (17.29%)	3.59	Salary Boost	Financial Constraints
		15–30 min	76 (28.57%)	3.53	Salary Boost	Poor Health
		30–60 min	111 (41.73%)	3.30	Salary Boost	Poor Health
		>1 h	33 (12.41%)	3.20	Salary Boost	Poor Health

In the questionnaire, in order to get more detailed SWB data of professional females, the survey team selected a sample of 17 professional females with representative answers to questions regarding family information, job information, and daily behavior attributes in the contact questionnaire as the in-depth research objects for in-depth interview from September to December 2021. Since people’s understanding and perception of happiness are mostly subjective, open and narrative interviews were conducted with minimal guidance from the interviewer and mainly focusing on the interviewees. After the interviewer had asked their question, the respondent was allowed to speak freely. Finally, the researchers extracted useful information from the answers of the interviewees and summarized them. Due to COVID-19, both offline and online interviews were conducted. For offline interviews, interviewees were contacted in advance to arrange a time to meet in a café. Online interviews with 10 participants were conducted by telephone. Interviews were recorded with the consent of all interviewees and after they had been informed that their private information will be kept anonymous and confidential. The 17 interview participants were coded (Interview 1, Interview 2. Interview 17), and their career types mainly covered: Civil Servants, Public Institution Staff Members, State-owned Enterprise Employees, Foreign Enterprise Employees, and Private Enterprise Employees. Professional females’ family attributes included single, married without children, and married with children. Table 2 presents the complete descriptive information of the interviewees.

**TABLE 2:** Basic information of the respondent.

Name	Age	Family attributes	Career type	Workplace experiences (year)^a^
Interview 1	35∼44	Married without children	FE employee	6
Interview 2	35∼44	Married without children	FE employee	10
Interview 3	19∼24	Single	FE employee	1
Interview 4	19∼24	Single	FE employee	2
Interview 5	35∼44	Single	FE employee	12
Interview 6	35∼44	Married with children	FE employee	13
Interview 7	25∼34	Single	PI Staff Member	2
Interview 8	25∼34	Single	PE employee	5
Interview 9	25∼34	Single	PI Staff Member	3
Interview 10	19∼24	Single	SoE employee	2
Interview 11	25∼34	Married without children	PE employee	4
Interview 12	25∼34	Single	SoE employee	3
Interview 13	25∼34	Single	Civil servants	3
Interview 14	45∼54	Married with children	PE employee	19
Interview 15	25∼34	Single	PE employee	3
Interview 16	25∼34	Single	PE employee	7
Interview 17	25∼34	Married without children	FE employee	5

*^a^Workplace experiences: The number of years that a professional female has worked since she had a formal job.*

## Results

### The Current State of Subjective Well-Being of Professional Females

#### The Life Satisfaction State

**FIGURE 3:**
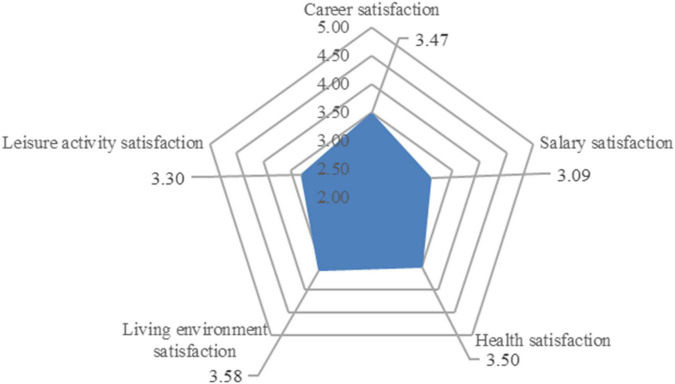
The life satisfaction score of professional females.

The five dimensions of professional females’ satisfaction with life were marked by a radar chart (Figure 3). Living environment satisfaction received the highest score (3.58), followed by health satisfaction (3.50), career satisfaction (3.47), leisure activity satisfaction (3.30), and salary satisfaction (3.09).

Professional females scored satisfaction with their residence the highest; in this survey, living environment satisfaction includes their comprehensive evaluation of their residence, community environment, and neighborhood relationship. Women are the leaders of domestic life, and they have greater decision-making power over the use of living place, allocation of related affairs in the house, and maintenance of the house. They also share a closer relationship with the community members around the house than men, and are more sensitive to the surrounding environment. Professional females in Dalian High-tech Zone had a higher evaluation of living environment than other affairs in their life. On the one hand, Dalian is the first city in China to be selected as one of the world’s 500 best cities for environment, and its greening rate and livable degree are second to none among Chinese cities. Residents’ happiness with city life consistently ranks among the highest in the country, and in recent years, it has been successively awarded titles such as “The 200 strong city of the world with charismas” and “China’s Happiest City,” which has attracted many young people, especially those from north and northeast China, who regard Dalian as a city for striving and pursuing their dreams.

Salary satisfaction was scored the lowest among the five life satisfaction categories of professional females, which shows that professional females are unhappy with their pay situation. Overall, the gender difference in salary still exists significantly in the urban employment environment. Dalian has long been known as a city of high consumption and low wages, especially for women, who in male-dominated industrial and information companies in High-tech Zones find it difficult to get promoted, secure pay raises, or even make a difference (Chen and Chen, 2021). For civil servants and staff of public institutions within the state-system, although the gender difference in basic salary is not significant, men are more able to take on overtime and difficult work, they are valued more by leaders, and their extra pay is slightly higher than that of women. Many professional females also face decreased job mobility, pay cuts, or even passive loss of promotion opportunities. Despite years of fighting for gender equality, gender differences in the workplace persist. Considerable evidence and research show that the hidden value created by women for society is greater than that by men, however, they receive a mismatched return. In addition, at work, they also take on more invisible unpaid work, which is the basis of social reproduction and continuation of human race. Therefore, among the five types of life satisfaction, professional females are the least satisfied with their salary.

**FIGURE 4:**
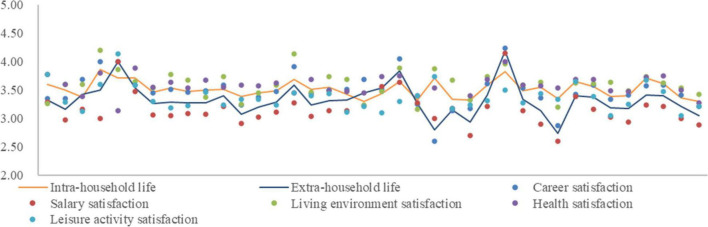
Life satisfaction of professional females based on various attributes.

There are great differences in the life satisfaction scores of professional females with different attributes (Figure 4), according to the sorting results of Table 1, each point in Figure 4 represents professional females of each attribute. Women with a monthly income of more than 10,000 yuan reported the highest career satisfaction, salary satisfaction, and health satisfaction; women aged 45–54 reported the highest satisfaction with their living environment; and women aged 55 and over were most satisfied with leisure activities. Female employees in private enterprise had the lowest job satisfaction, and those with working hours > 8 h had the lowest salary satisfaction. Female employees in foreign enterprises had the lowest satisfaction with their living environment. Women aged over 55 were least satisfied with their health, and women with a commuting distance of 5–10 km had the lowest satisfaction with leisure activities.

Integrating the five categories of life satisfaction into intra- and extra-household, women aged 35–44 and over 55, civil servants, public institution staff members, employees of state-owned enterprises, and women with monthly income of more than 10,000 RMB had higher life satisfaction in intra- than extra-household life, and other types of women had higher life satisfaction in extra- than intra-household life.

In addition, for professional females aged 35–44, working in foreign-owned companies, with family vehicle ownership ≥ 2, and married with children, their scores of life satisfaction in intra- and extra-household life were close (difference of less than 0.1). While for those in private enterprises and working 5–8 h or above 8 h, there was a significant difference in their scores of life satisfaction in intra- and extra-household life.

#### The Emotional Cognition State

**FIGURE 5:**
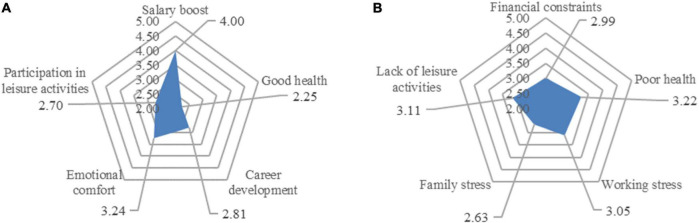
The emotional cognition score of professional females.

The radar chart was also used to show the overall sources of positive and negative emotions of professional females (Figure 5). First, regarding the scores of the two emotional sources, professional females’ positive emotional sources were ranked as: 4.00 for salary boost, 3.24 for emotional comfort, 2.81 for career development, 2.70 for participation in leisure activities, and 2.25 for good health; their negative emotional sources were ranked as: 2.63 for family stress, 2.99 for financial constraints, 3.05 for working stress, 3.11 for lack of leisure activities, and 3.22 for poor health.

Comparing the scores of positive and negative emotion sources and the two radar maps, it can be observed that the corresponding supplement of positive and negative emotional sources of professional females is not consistent. Salary boost for them was the main source of positive emotions, but financial constraints was not their main source of negative emotions, instead, it was poor health. Conversely, good health was the least significant source of positive emotions. This suggests that an increase in income makes professional females feel the happiest, however, financial constraints have no significant effect on their SWB, while poor health was the most depressing factor for them. From the perspective of leisure activity participation, it did not significantly increase the positive emotions of professional females, but lack of leisure activities was the second biggest source of negative feelings among them. This shows that leisure activities, which regulate individual emotions, are indispensable for professional females and have become a part of their life, and if they lack leisure activities, they will obviously feel depressed.

**FIGURE 6:**
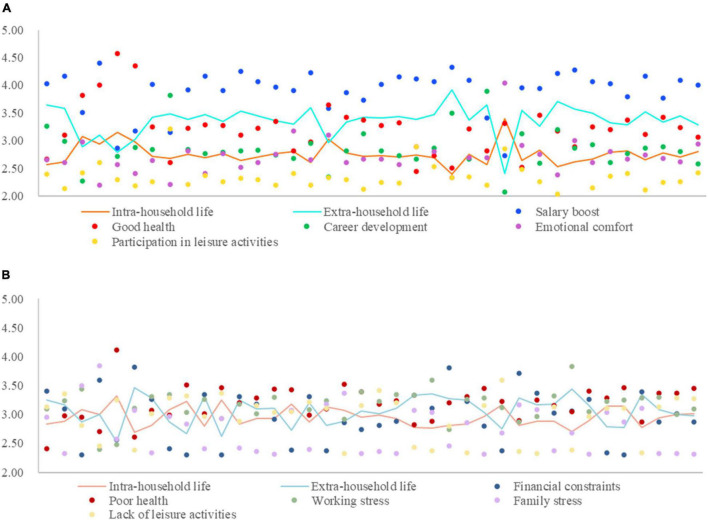
Emotional cognition of professional females based on various attributes. **(A)** The sources of positive emotions. **(B)** The sources of negative emotions.

Figure 6 presents the sources of positive and negative emotions of professional women based on various attributes. The sources of positive and negative emotions with different attributes were quite varied, especially the difference between intra- and extra-household life.

The source of positive emotion is consistent with the result of the radar map, which is mainly derived from salary boost. For females aged 35–44 and above 55, with an education of high school and below, and married with children, their primary source of positive emotions was good health. For professional females with a masters’ degree or above and monthly income of 5 001∼10 000 RMB, their primary source of positive emotion was career development. The primary source of positive emotion for professional females with monthly income > 10,000 RMB was emotional comfort. An additional primary source was salary boost. Thus, in the life of extra-household, financial incentives are the most stimulating for professional females. In terms of age, based on the results of the survey, among those aged 35–44, 91.04% were civil servants and employed in public institutions and foreign companies; these jobs are more stable than, say, the private sector, hence, the women working in such organizations have been enjoying a relatively stable family life and work. However, their salary would not have increased significantly in the current phase, and they would be supporting the family together with their husbands; thus, an increase in income did not significantly increase their positive emotions. However, as 77.61% of these women were married and 86.54% of them had children, they had to take care of their families and children while dealing with work tasks, hence, the dual pressure of work and family requires them to be in good health. Professional females over 55 years old are gradually entering old age. They are about to retire and with increasing age, the maintenance of health has become an important task for them. Professional females with a master’s degree or above and monthly income of 5,001∼10,000 RMB pay more attention to their career development due to their higher education level and their career development status, thus, their positive emotions are mainly based on career development. Combined with the life satisfaction score, the overall life satisfaction of professional females with monthly income > 10,000 RMB was 3.97, the highest score of all. Their other life satisfaction scores were also high. As their material world is more abundant, their main source of positive emotions is emotional comfort.

By integrating these five sources of positive emotions into intra- and extra-household life, expect for women aged 35∼44 and above 55, married childless, and with monthly income > 10,000 RMB, the positive emotions of other professional females mainly come from extra-household life. The ranking of five types of negative emotions from domestic and external sources are in the state of high and low penetration. Different types of professional females reported different primary sources of negative emotions, among them, poor health and working stress were the main sources of negative emotions, while family stress and lack of leisure activities were the least significant sources of negative emotions.

### Influencing Factors of Professional Females’ Subjective Well-Being

#### Influencing Factors of Life Satisfaction

**FIGURE 7:**
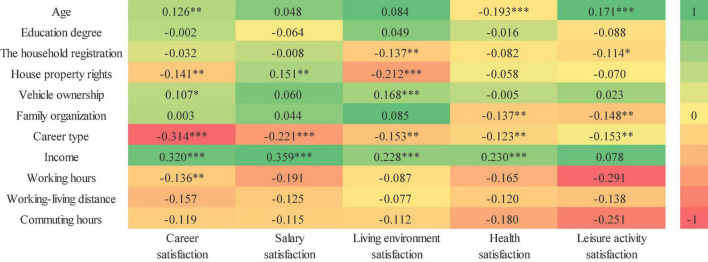
Spearman correlation values of five types of satisfaction. “^***^” Means significant at the 0.01 level; “^**^” means significant at the 0.05 level; “*” means significant at the 0.1 level.

Spearman’s correlation was used to analyze the correlation between various attributes of professional females and life satisfaction. It is a method to study the correlation between two variables based on grade data, calculated according to the difference of equal series of two pairs of grades, so it is also called “grade difference method.” According to the principle that either of the two columns of variables is a rank variable, when analyzing the influencing factors of females’ attributes and life satisfaction, by conducting pairwise correlation analysis of each variable. The attributes of each woman and their scores on five life satisfaction items were integrated into a data set and imported into SPSS, with the results visualized by color level diagram (Figure 7).

The correlation coefficient between career satisfaction and age (β = 0.126, *p* < 0.05), house property rights (β = –0.141, *p* < 0.05), vehicle ownership (β = 0.107, *p* < 0.1), career type (β = –0.314, *p* < 0.01), income (β = 0.320, *p* < 0.01), and working hours (β = –0.136, *p* < 0.05) was significant. Among these, career satisfaction was positively correlated with age, vehicle ownership, and income. It was negatively correlated with house property rights, career type, and working hours. Career satisfaction was positively correlated with age and income, but negatively correlated with career type. As professional females grow older, their work situation becomes more stable, especially after many years of continuous work in the same position, their seniority is gradually accumulated. In particular, civil servants and public institution staff members at the post and technical levels are promoted according to their length of service, with their income also increased. Consequently, women in both occupations reported higher life satisfaction. However, most women working in private enterprises think that such organizations have low stability, high requirements for age, and rapid dismissal of employees, hence, their career satisfaction is relatively low.

Salary satisfaction was significantly correlated with house property rights, career type, and income. It was positively correlated with house property rights (β = 0.151, *p* < 0.05) and income (β = 0.359, *p* < 0.01), and negatively correlated with career type (β = –0.221, *p* < 0.01). The relationship between salary satisfaction and career type and income of professional females was similar to that of career satisfaction, however, its relationship with home ownership was contrary to that of career satisfaction. This means that women who rent are more satisfied with their pay than those who own a house. This is because for young people in modern China, once they have a stable job, it is time to start buying a house, however, China’s housing prices have remained stubbornly high, especially in the first- and second-tier cities. Dalian is among the top cities in northeast China in terms of economic development, characterized with high housing prices and consumption levels. Therefore, after buying a house, professional females face more economic pressure and lower salary satisfaction.

Living environment satisfaction was significantly and positively correlated with vehicle ownership (β = 0.168, *p* < 0.01) and income (β = 0.228, *p* < 0.01), and negatively correlated with household registration (β = –0.137, *p* < 0.05), house property rights (β = –0.212, *p* < 0.01), and career type (β = –0.153, *p* < 0.01). Professional females with higher economic attributes, such as income and family car ownership, are situated on a higher living environment level, therefore, they are more satisfied with their living environment. The living level of women with non-local household registration in Dalian was relatively poor compared with that of women living in rented houses, which results in low satisfaction of living environment. After entering the system, professional females who are civil servants and employed in public institutions and state-owned enterprises were more inclined to buy houses in Dalian, so their satisfaction of living environment was higher than that of women who work in foreign and private enterprises.

The correlation coefficient between health satisfaction and age (β = –0.193, *p* < 0.01), family organization (β = –0.137, *p* < 0.05), career type (β = –0.123, *p* < 0.05), and income (β = 0.230, *p* < 0.01) was significant. Among these, in addition to income, health satisfaction was negatively correlated with age, family organization, and career type. As women get older, their health satisfaction declines, and after building a family, the arrival of children also affects the health of professional females.

Leisure activity satisfaction was significantly correlated with age (β = –0.171, *p* < 0.01), household registration (β = –0.114, *p* < 0.1), family organization (β = –0.148, *p* < 0.05), and career type (β = –0.153, *p* < 0.05). All these attributes were negatively correlated with leisure activity satisfaction. The workload of professional females tends to decrease as they get older. Consequently, their leisure time increases and their leisure activity satisfaction is higher. However, women who have children after marriage spend most of their leisure time taking care of the family and children, so they are less satisfied with leisure activities.

#### Influencing Factors of Emotional Cognition

(1) Sources of positive emotions in everyday life

“home” as important “pockets of local order” (Lenntorp, 2004) for residents’ daily activities, and for professional females, it is the destination of professional female’s spatial behavior. Therefore, in intra-household life, they feel happy when they meet with their parents and family. While for married professional females, having a happy marriage entails having no children and free time and enjoying the state of two-person world with their husbands.

*“My hometown is not in Dalian, I finished my university study in Dalian, after graduating, I got my present job in Dalian, so I have been staying in Dalian, but no matter how busy I am, I call my parents after work every night, it’s relaxing for me to chat with them, and I also seek my parents’ advice when I encounter difficulties in my daily life, my hometown is far away from Dalian, I can’t go back there to see my parents on weekends, but I will go back during the mini-break.”*

*“My husband and I have been married for years, but we never thought about having children, and we still don’t, because the two of us live very freely and are comfortable with the current pattern, neither of us wants to change, and we want to spend more time with our parents, and if we have children, our leisure time after work will be completely around them, and our parents will have to help us look after them, they will be tired.”*

*“My husband and I have just been married for a year. We love each other very much and he does all the housework, our marital house is also based on the location of my work place, it is convenient for me to commute. We have free time after work, it gives us a lot of time to travel. Because we are still young, we are not planning to have children for a few years and we are enjoying being alone for now.”*

Although caring for children can be stressful for women, the companionship of children can also make them happy, and intergenerational care reduces the burden on married professional females with children:

*“Although I have no private life since the birth of my daughters and devote my spare time to my family, I still feel happy seeing my children and watching them grow up happily every day. My husband and I take our two daughters out for trip every holiday season, this joy of parenthood I had never experienced before marriage or childbirth.”*

*“Since I gave birth to my daughter, I have felt my life as a woman is complete. Watching her grow up day by day, passing on my life experience and life principles to her, enabling her to receive better education and train her to become a better person is the most fulfilling thing in my life.”*

*“I have two children. One is in primary school and the other is in kindergarten, I spend my leisure time at home with my kids. Because my husband is busy at work, I do more of the childcare, but my parents also live in Dalian, so they help me take care of my children, as my mother is responsible for driving the children to and from school and cooking for them, it helps me a lot.”*

In addition to family, professional females also enjoy spending time with friends, hanging out with friends is an important way for professional females to relieve stress and relax:

*“When I take a break from work, I go out with my friends and participate in activities, such as hiking, climbing, shopping, and so on. The reason I don’t talk to my family when I’m in trouble is because they will be worried, and they won’t understand the pressures I encounter at work, but I talk to my friends, we understand each other better.”*

Career satisfaction is the most important source of happiness for professional females in extra-household life:

*“I am a geography teacher in institutional school, I like geography very much. I majored in geography during my undergraduate and master’s years, teaching has always been my ideal job, therefore, I chose to be a geography teacher in high school after graduation, I enjoy and am satisfied with my present job, and it’s a job within the state system, the salary and treatment are good, and the social status is high. I am also very grateful to the city of Dalian, because it has given me an ideal career and considerable income.”*

*“I was admitted to the civil service after my master’s degree, I’m a treasurer for a county government, although it’s a tough job, I can handle it, because this job is exactly in line with my bachelor’s and master’s major, I’m proficient at it. Both my parents and I are satisfied with my job because the social status is high and the job is stable.”*

*“I joined my current company after I graduated from master’s degree, which is a German enterprise, I’m an assistant to the general manager in the company. I really like the relaxed working atmosphere and open enterprise culture in foreign companies, the company’s work task allocation is very careful, what I need to do is my own work, there is no need to work overtime, and it is not recommended or encouraged, and I don’t have to worry about work during the holidays, and I can completely disappear into thin air, so I have a lot of free time after work.”*

As a result, the more educated a woman is, the more likely she is to find a satisfying job, consequently, career satisfaction, salary satisfaction, and other life satisfaction in extra-household life will be higher, and higher welfare benefits in the workplace also have a positive impact on the happiness of professional females:

*“I have been working for this Japanese company for over ten years. Many holidays in Japanese companies are arranged according to the Japanese solar terms, and in Japan, there are many solar terms and long holidays, such as “Red days,” “Old People’s Day,” and so on, and to catch up with Chinese traditional festivals, we also have holidays or paid holidays, and the company never requires or encourages employees to work overtime, so I have a lot of leisure time after work.”*

*“There are a lot of hidden benefits that companies provide us, for example, our company has a special “plant doctor,” including some professional psychologists, so when I encounter pressure at work, I often seek help from psychological doctors, which is of great help to relieve my pressure.”*

Foreign companies do not encourage overtime, have fixed commuting time, and provide abundant hidden benefits to their employees, especially women, who receive paid post-marriage leave and are allowed to leave work 1-h early after the birth of their child, in addition to the legal maternity leave. Many foreign companies also arrange free medical check-ups for female employees every year, and have separate dressing and maternity rooms for female employees. Some large foreign companies also use the slogan “Women welcome” to encourage women to apply. Further, women are not asked about their personal lives and marital status during the recruitment process, and the company’s leadership is equally divided between the sexes:

*“My department manager is male, and the department manager parallel to my department is female.”*

(2) Sources of negative emotions in everyday life

Marriage and childbirth are important key events in a woman’s life (Yuqing et al., 2021). Single professional females are pressured “to get married,” and married professional females are pressured “to have children”:

*‘‘Although my parents and I live in the same city, we don’t live together, I’ll go back to see them during the holidays, they make me delicious food. But every time I came home, my father would urge me to find someone and get married as soon as possible, it gets on my nerves every time, and family around me often set me up on blind dates^1^, I’ve already met seven or eight blind dates this year, but none of them is my type, and I’m tired of seeing them again, but the fear of missing the ‘right fate,’ torments me.”*

*“I am currently newly married, childless, live freely with my husband, my husband is a college teacher, we plan to go to the United Kingdom together next year, he will go on an academic visit and I will accompany him, and I will probably quit my job and apply for a master’s degree in the university where he will be visiting in the United Kingdom. Our parents said they’re supportive, but every now and then they ask us: When are we going to have a child? They hope we have children early, in their current age and physical condition, they can help us take care of children. But my husband and I have no plans to have children in the next few years. We have a lot of work to do, so sometimes we get tired of what our parents say.”*

Participants were not only pressurized by the parents, but also themselves harbored the expectation and longing to get married and have children:

*“I am single and lonely now, so I hope to start a family, I was desperate for love, but there were few opportunities to meet other boys and few single men my age at work.”*

*“When I graduated from college, I set myself the goal of getting married by 27 and having kids by 30, but now I’m approaching the age where neither goal has been achieved, so it bothers me.”*

*“My husband and I have been childless since our marriage, so we are very free after work, we can go out whenever we want, but actually we always wanted to have a baby, and since we got married late, I was worried that we weren’t physically fit enough to have children, so we spent the last few years trying to adjust our bodies to get pregnant.”*

The traditional concept of “man goes out to work while woman looks after the house” still permeates the domestic life of modern professional females:

*“When we have guests, I do most of the entertaining, including cooking and household hygiene. Although our generation has made a lot more progress in terms of gender equality than the older generation, but sometimes when my parents come to visit us and find a mess, they think that I did something wrong that I didn’t do my duty as a wife. So sometimes I still feel unhappy and wronged, and my parents would tell me: you should cook often for your husband in your spare time, which will have a warm family feeling, and it’s good for your health.”*

Working stress is the main source of negative emotion of professional females’ extra-household life, and the restriction of working time on life can cause serious health problems for working women, including lack of exercise and unhealthy diet, which can seriously affect their SWB:

*“Overtime work is very serious in our company, my normal working hours are 10:00 – 19:00, although we have a flexible work schedule, we can arrive at the office by 10:30 at the latest, it also led to longer working hours, because the company leaders require us to leave work at least around 21:00 in the evening, it is normal for company leaders to work overtime ‘appropriately.’ And because we are facing an impending performance appraisal, everyone will work overtime under such pressure, so we all get tired, and working in an emotional state is not productive. Even when I come home from work, I have to respond to messages and be ready to solve problems whenever there is unfinished work or anything I need to do, which makes it impossible for me to really relax and get some rest.”*

*“Due to the pressure of work, I have been diagnosed with a lot of diseases in recent years, and a lot of money has been spent on hospital treatment. Therefore, after several years of work experience, I think health is the most important.”*

*“I bought my own house in the last two years, so I had to scrimp and save to pay the mortgage, and because of my increased age, many tasks at work are not as handy as they used to be. Due to all kinds of pressure, my health has not been good in recent years. I often had to go to the hospital, buy medicine and care products, and spend a lot of money on them.”*

*“The most depressing thing in my life right now is that I want to stay healthy, I want to go to the gym and do more exercise to keep fit, but I don’t have the time; I want to eat healthier and regularly, but I don’t have time to cook for myself and have to order take-out a lot.”*

It can be seen, there is a big difference in the flexible work schedule of domestic and foreign companies. The latter’s working system requires employees to complete their work during their working hours, and there exists a clear boundary between work and rest. However, in domestic enterprises, although employees are allowed to be a little late for work and take a little more rest during lunch break, the work continues to seep through the personal life of employees even after work hours.

Apart from the pressure of working hours and content, interpersonal problems in the workplace can also cause negative emotions for professional females:

*“The most stressful thing for me is the busy work, high work intensity, and less time for rest. Recently, an old accountant just retired from our company, and I took over all her work, which has added another layer of work commitments on top of my own work. However, due to the poor relationship between us, she did not arrange the handover work with me before she left, which means I had no way to get started.”*

*“I am preparing for the intermediate accountant certificate. Every time when I review my exam material in my spare time, a senior colleague in the company who already has the intermediate accountant certificate will interfere with my review. She is worried that I would affect her future career, which makes things awkward between us.”*

*“I often have some friction with my leader, the colleagues in my office often give her some small gifts or praise her, while I am a straightforward person, I never pretend to get along with her, so she does not pay much attention to me, causing me to miss out on a lot of development opportunities.”*

In traditional Chinese interpersonal communication, people care about the worldly wisdom and reciprocity of courtesy, especially in an institutional work environment, “guanxi” is an important way to maintain professional status. This includes two aspects: one is whether individuals can have a good interpersonal relationship with their leaders or colleagues and be welcomed by others; the second is whether they have a network of relationships to ensure that someone helps them in their work and prevents them from being bullied by others.

However, in a foreign enterprise’s work environment, everyone is more self-centered:

*“I work with a lot of foreigners from different countries and cultures, such as Germans, Austrians, Canadians, and so on, we often have problems communicating and understanding, for example, if I organize a meeting, I want to complete five tasks in that meeting, but due to different understandings, they often go off topic or fail to complete the tasks in the meeting.”*

Gender inequality at work can significantly lower the mood of professional females, outside, even professional females themselves attribute gender inequality to biological differences between men and women:

*“Men get more opportunities at work, and are valued more by the bosses than women, especially when it comes to pay and promotions. For example, a male colleague entered at the same time as me, we were in the same class in the same school for our master’s degree, we were all on the same starting point, and I was the monitor at that time, but he was obviously more valued by the leaders, of course I feel unhappy, and sometimes it affects my mood at work.”*

*“I’ve seen a lot of gender inequality in the workplace. First, when I applied for this company, it was obvious that the company preferred male candidates. Second, when going on business trips or connecting with other enterprises, leaders prefer to send male colleagues, believing that they can better socialize with other enterprise personnel, men are thought to be better able to tolerate long business trips.”*

*“Under the same academic background, the male employees around me have more room for advancement and faster promotion than the female employees, because men are able to work under greater intensity and stress than women. For me personally, including myself, women are no less competent than men, but men are indeed doing better at resisting stress than women. For example, when my unit is busy, male colleagues are more likely to stay up late and recover faster physically and psychologically after fatigue than female colleagues. Also, women spend more time on their personal lives than men. For example, compared to my male colleagues, I spend more time on personal hygiene and grooming before going to work each day, which virtually reduces my time for work and rest.”*

Professional females’ anxiety about age and ability seriously affects their mood. They often feel uncertain of the future and fear being eliminated at work:

*“I went to university in Dalian and started to work here right after graduation. Now I feel that my level is too low and I can only accept some simple jobs with insufficient education background, so I want to improve myself. I attended an international trade training class last year, hoping to improve myself in related fields and get some qualifications. In addition, as my job is related to Japanese, I will continue to learn Japanese at ordinary times. Although weekends and holidays are normal, I still don’t have a large chunk of continuous time to study, and the mindset has changed from when I was in college, now the things I want are very realistic, and when I experience work pressure or my emotional state is low, I am in no mood to learn.*

*“Due to the macroeconomic downturn caused by the COVID-19 pandemic in recent years, the company is considering staff reductions and redeployments, which worries me. Because I am satisfied with the current working environment and work content, I am afraid that I will be transferred to another department. My job is BPO, the work is the same every day, and it is relatively simple, less technical, and does not reflect personal value, so I was afraid that I would be eliminated. In addition, as the labor price in China is getting higher and higher, foreign software outsourcing is less inclined to choose China, but will turn to Thailand, India, Cambodia, and other Southeast Asian countries, so the workload received by our company in recent years has decreased significantly.”*

Many professional females desire to increase their self-worth, and those working outside the system actively seek opportunities to work inside the system:

*“In the near future, my goal is to review the relevant content of the intermediate accounting qualification certificate carefully in my spare time, and strive to obtain the qualification certificate next year, to improve my working ability. My long-term goal is to start my own business and open an accounting firm.”*

*“Although my current job is ok and not very tiresome, it is a private company after all, and I still have a feeling of instability. In addition, many of my friends have been admitted into the system in the past few years, so I also actively look for opportunities to apply for the right institution in the system, and will start to review after setting goals in the next one or two years.”*

## Discussion

In the context of the post-modern trend that puts people first and emphasizes differences, feminist geography has gradually become well-known as an emerging discipline that cares about women and minorities. Owing to research on the characteristics of temporal and spatial activities (Fernandez and Su, 2004; Mcdowell et al., 2006; Crane, 2007) and behavioral patterns of residents (Schwanen et al., 2008; Hanson, 2010; Chung and Hahn, 2020) based on a feminist perspective, the study of psychological activities and emotional experiences of different female groups (Kang and Scott, 2008) has made great progress. Since the founding of the People’s Republic of China, equality between men and women has always been a basic national policy. “Women’s liberation” has been integrated with “class liberation,” “national liberation,” and “national rejuvenation,” with various laws and regulations clearly stipulating provisions to protect women’s rights and interests. With the development of social economy, today’s Chinese women are more independent, their educational level and labor force participation rate are equal to those of men, and some have even surpassed men in many fields, causing work to gradually become the main theme in their lives. The research on professional females in Chinese geography and sociology has become more diversified. Many empirical studies have found that women cannot be separated from the traditional Chinese family model of “man goes out to work while woman looks after the house” while dealing with work tasks (He et al., 2017), especially married women and women with children. The dual burden of family and career has a negative impact on women’s life satisfaction and mental health (Wu et al., 2015). Research shows that the current marriage rate in China is decreasing, and the marrying age of women is increasing. Although China holds a large population, it is currently facing a severe situation of an aging population and a declining birthrate; moreover, the willingness and number of women who plan to have children are both declining, thus, the fertility policy is constantly being adjusted. The discussion on these issues is worth being carried out from the perspective of subjective feelings of women toward their daily life. Therefore, taking Dalian High-tech Industrial Zone as case area, based on the dual constraints of intra- and extra-household life of professional females in feminist geography, this study analyzes the SWB of contemporary Chinese professional females.

Regarding the research background, first, due to the implementation of the unit system after the liberation of China, female groups receive special treatment from the employment and childbirth welfare systems (Tianbao and Yanwei, 2012). Dalian is situated in northeast China’s Liaoning Province, which in the Planned Economy Period constituted one of the three provinces in northeast China known as the “eldest son of the Republic.” Due to the construction of heavy industrial bases followed by a high level of urbanization, the unit system referred to as the “full employment system” encouraged urban women to step out of the house and enter the society as a system. During this era, most urban women obtained permanent jobs from their affiliated organizations (Aihua, 2001). However, with the gradual disintegration of the unit system and the establishment of a market economy, the gender gap in Chinese cities is gradually expanding, as reflected in all aspects of social life, such as employment, income, and family division of labor. The invisible discrimination faced by women in the workplace is also manifest (Lin et al., 2020). Second, due to the unique lifestyle of northeast China that experiences cold climatic conditions and is inspired by the saying “planting in spring, harvesting in autumn and relaxing in winter,” women who dominated domestic life had a greater say in family life during the slack farming period. The continuation of this traditional way of life has also led to the higher status of women in northeast China. Third, dual-employee families in Chinese cities are the mainstream of urban society. The ratio of dual employees here is far greater than that in Western cities, with women being responsible for the management and execution of most things at home (Feng et al., 2013). Especially after childbirth, the pressure of parenting increases the burden on women, and many professional females even choose to leave the workplace. The implementation of the current three-child policy not only increases the expectations of the society and families concerning childbirth, but also deepens the discrimination against professional females in the workplace. In a society with intergenerational relationship, older adults, after retirement, help take care of their grandchildren (Maurer-Fazio et al., 2021). This reduces the parenting burden of professional females to a certain extent, but taking care of older parents also becomes their future responsibility (Goh, 2009; Feng et al., 2015). These abovementioned burdens together constitute the dual dilemma of work and family faced by professional females, greatly reducing their SWB. China has promulgated many laws and policies to protect the rights and interests of professional females to reduce their daily pressures and promote employment. For instance, both the “Special Provisions on Labor Protection for Female Employees” promulgated by the State Council and the provincial “Regulations on Population and Family Planning” stipulate the maternity leave regulations for professional females, mandating that they “enjoy 98 days of maternity leave during childbirth, including 15 days of leave before childbirth.” The maternity insurance stipulated by the Ministry of Human Resources and Social Security of China includes both maternity medical expenses and maternity allowances. Employers must provide maternity fees even if they do not pay maternity insurance. Likewise, the benefits of maternity insurance will no longer be limited to household registration, aiming to provide professional females with maternity allowances, medical services, and maternity leave to help them regain their working capacity and return to work. The “Guiding Opinions on Promoting the Development of Care Services for Infants and Children Under 3 Years of Age” issued by the State Council in 2019 also gradually explore the implementation of China’s parental leave. China’s current retirement age is 60 for men, 55 for female cadres, and 50 for female workers. These policies take into account women’s internal differences and aim to reduce their workload. However, they are implemented to different degrees and have different effects in different workplaces. For example, female employees working in foreign and private enterprises hope to work in the system to obtain more stable welfare and future pension security. While the female employees working in the system face more depressing psychological problems such as gender inequality in promotion and career development, interpersonal relationship handling, and unpaid overtime. Therefore, the social development background that affects professional females’ SWB is worthy of further study in the future.

Regarding research participants, in addition to several types of professional females included in this study, there are large number of women engaged in informal employment. This mode of employment allows women to participate in economic activities while balancing their traditional family roles, thus, providing an effective channel for them to generate income to alleviate poverty. It is also beneficial in enhancing women’s family status and changing the traditional gender relations to some extent. However, such female labor tends to be at the lower end of the labor market and marginalized, where employers do not focus on developing their skills. Therefore, these women lack self-consciousness of upward development and are unable or difficult to achieve formal employment by mastering new skills and improving their human capital (Yanhan and Desheng, 2009; Gengzhi et al., 2011). Therefore, their SWB is also worth exploring. In addition, the measurement and evaluation of SWB of women in different age groups, social classes, and regions are also worth further discussion in the context of the increasing internal differences among women in Chinese society, in order to depict the actual living conditions of contemporary women from a more comprehensive and rational perspective, enrich the research framework of feminist geography, and support the development of women in all aspects.

## Conclusion

This study conducted a field survey on professional females in Dalian High-tech Industrial Zone, used questionnaire survey and in-depth interview data, and combined their intra- and extra-household life, to analyze the SWB of professional females from the perspectives of life satisfaction and emotional cognition.

Quantitative research results revealed the following: (1) the life satisfaction of professional females is ranked from high to low, as follows: living environment satisfaction > health satisfaction > career satisfaction > leisure activity satisfaction > salary satisfaction. For most, their life satisfaction intra-household was higher than that extra-household. (2) Regarding emotional cognition, professional females rated their sources of positive emotions from highest to lowest, as follows: salary boost > emotional comfort > career development > participation in leisure activities > good health; their sources of negative emotions from highest to lowest were: family stress > financial constraints > working stress > lack of leisure activities > poor health. (3) Spearman’s correlation was used to analyze the correlation between professional females’ various attributes and life satisfaction, which varied with individual attributes, reflecting the internal differences among them, because the objects of the survey included not only civil servants and staff members of public institutions working in the national system, but also female employees working in state-owned, foreign, and private enterprises. Moreover, as the work content and family and personal attributes of professional females in different career types vary greatly, the factors affecting their life satisfaction are also different. Therefore, we selected 17 representative respondents and conducted in-depth interviews with them, to extract the sources that influence professional females’ emotional cognition from the perspective of individual differences.

From this, the qualitative research results showed that, first, the sources of professional females’ positive emotional cognition were as follows: (1) In intra-household life, they included the company of parents, family, and friends; a free life with the husband in a childless marriage; and after having children, in the process of seeing their children grow up. (2) In extra-household life, they included their satisfaction with the working environment and treatment. Second, the sources of negative emotions were: (1) Pressures of “marriage” and “birth” and the traditional concept of “man goes out to work while woman looks after the house” in intra-household life. (2) Health problems caused by working stress, interpersonal problems and gender inequality in the workplace, the anxiety of age, self-improvement, and future career development in extra-household life.

According to these study results, the status of contemporary Chinese women in family life has improved significantly, but gender inequality in family life and workplace continues to exist. The daily life of professional females is still family-oriented, they enjoy the company of families and friends, but are still influenced by some traditional ideas. In the social field, professional females still face various forms of explicit or implicit discrimination and gender inequality, which significantly reduce their enthusiasm and happiness at work, even affecting their health. However, it is also clear that they still live a positive life and strive to improve their self-worth, which reflects the bravery, confidence, and beauty of contemporary Chinese women, in keeping with the trend of the times.

## Data Availability Statement

The original contributions presented in this study are included in the article/supplementary material, further inquiries can be directed to the corresponding author.

## Ethics Statement

Ethical review and approval was not required for the study on human participants in accordance with the local legislation and institutional requirements. Written informed consent for participation was not required for this study in accordance with the national legislation and the institutional requirements.

## Author Contributions

YZ: conceptualization and methodology. YG and CZ: investigation and writing – original draft. TL and XL: supervision and review and editing. All authors contributed to the article and approved the submitted version.

## Conflict of Interest

The authors declare that the research was conducted in the absence of any commercial or financial relationships that could be construed as a potential conflict of interest.

## Publisher’s Note

All claims expressed in this article are solely those of the authors and do not necessarily represent those of their affiliated organizations, or those of the publisher, the editors and the reviewers. Any product that may be evaluated in this article, or claim that may be made by its manufacturer, is not guaranteed or endorsed by the publisher.

## Footnotes

1 Blind dates: It is a kind of traditional Chinese marriage custom and wedding etiquette. In ancient times, it was arranged by matchmakers and parents. This way of choosing a mate has continued into modern times, but it has changed, unlike in ancient times, matchmaking only emphasized the importance of “matching family background” and followed the “order of parents and matchmaker’s words.” In the process of contemporary Chinese blind dating, young people will check whether the blind date is suitable for them through appearance, family condition, occupation, social status, especially the subjective feeling, to determine whether to move forward with the relationship.
